# Routine data underestimates the incidence of first-line antiretroviral drug discontinuations due to adverse drug reactions: Observational study in two South African cohorts

**DOI:** 10.1371/journal.pone.0203530

**Published:** 2018-09-05

**Authors:** Reneé de Waal, Karen Cohen, Andrew Boulle, Matthew P. Fox, Gary Maartens, Ehimario U. Igumbor, Mary-Ann Davies

**Affiliations:** 1 Centre for Infectious Disease Epidemiology and Research, School of Public Health and Family Medicine, University of Cape Town, Cape Town, South Africa; 2 Division of Clinical Pharmacology, Department of Medicine, University of Cape Town, Cape Town, South Africa; 3 Boston University Departments of Epidemiology and Global Health, Boston, MA, United States of America; 4 Health Economics and Epidemiology Research Office, Department of Internal Medicine, School of Clinical Medicine, University of the Witwatersrand, Johannesburg, South Africa; 5 US Centers for Disease Control and Prevention, Division of Global HIV & TB, Pretoria, South Africa; University of Oxford, UNITED KINGDOM

## Abstract

**Introduction:**

Estimating the incidence of antiretroviral discontinuations due to adverse drug reactions (ADRs) is important to inform antiretroviral treatment (ART) regimen recommendations, and to guide prescribing and monitoring policies. Routinely collected clinical data is a useful source of pharmacovigilance data. We estimated the incidences of first-line antiretroviral discontinuations due to ADRs using routine clinical data, and compared them with incidences estimated using data enhanced by folder review, in two South African cohorts.

**Methods:**

We included patients 16 years and older on first-line ART. We selected a stratified random sample of 25% for checking of ART prescription data and reasons for antiretroviral discontinuations retrospectively, including folders reviews where required (enhanced-data sample). We estimated the incidence of antiretroviral discontinuations using Kaplan-Meier and competing risk analyses.

**Results:**

In 15396 patients, 40% had a first-line antiretroviral discontinuation by three years on ART. We could determine the reason for 65% of discontinuations using routine data only, and 84% of discontinuations, in the enhanced-data sample of 3837 patients. ADR was the most common reason for discontinuations. In the enhanced-data sample, the cumulative incidence of discontinuations due to ADRs by three years was 30.4% (95% CI: 24.4–36.6) for stavudine; 2.0% (95% CI: 1.5–2.6) for tenofovir, and 1.3% (95% CI: 0.8–2.1) for efavirenz. Using routine data only, the cumulative incidences of discontinuations due to ADRs by three years for stavudine, tenofovir, and efavirenz respectively were 23.9% (95% CI: 20.3–27.7), 1.2% (95% CI: 0.9–1.4) and 0.5% (95% CI: 0.3–0.7).

**Conclusions:**

Although the relative rankings were similar using routine or enhanced data, lack of checking for missing reasons for discontinuation resulted in underestimates of the incidence of antiretroviral discontinuations due to ADRs. Systems to improve data collection of reasons for regimen changes prospectively would increase the capacity of routine data to answer pharmacovigilance questions.

## Introduction

Adverse drug reactions (ADRs) caused by antiretroviral drugs can cause substantial morbidity and can result in compromised adherence to treatment.[1] In order to minimise potential harm to patients, it is important to determine the incidence, nature of, and risk factors for antiretroviral ADRs in routine clinical practice. Local data on drug-specific treatment-limiting ADRs is needed to ensure that recommended antiretroviral regimens and monitoring policies are appropriate, and to raise awareness amongst clinicians. Establishing cohorts specifically to monitor antiretroviral ADRs has been recommended,[2, 3] but is expensive and time consuming. Existing observational programme-based cohorts have been suggested as a good source of pharmacovigilance data, particularly in resource-limited settings.[4] Observational cohorts often do not routinely collect detailed data on all ADRs, but treatment discontinuations or modifications are more likely to be recorded. However, the reason for treatment discontinuation or modification may not be routinely recorded. Estimating the incidence and risk factors specifically for discontinuations due to ADRs requires establishing the reason for antiretroviral discontinuations.

Several previous African studies have used routine cohort data to describe antiretroviral discontinuations due to ADRs.[5–9] In all cases, routine data collection systems were augmented by folder review in order to establish reasons for antiretroviral discontinuations. Most of the studies took place before the 2010 World Health Organization guideline change that replaced stavudine with zidovudine or tenofovir as part of the preferred first-line ART regimen and some of these were influential in changing ART prescribing policy.[8] One South African study conducted after the guideline change showed that discontinuations due to ADRs were much lower with tenofovir, rather than stavudine.[9]

We estimated the drug-specific incidence of first-line antiretroviral discontinuations due to ADRs using routine clinical data from two large South African cohorts. We compared the incidences with those estimated in a sample of patients where we checked reasons for antiretroviral discontinuations retrospectively. We also identified risk factors for antiretroviral discontinuations due to ADRs.

## Methods

### Inclusion criteria and sites

We included all antiretroviral-naïve patients who were at least 16 years old and started antiretroviral treatment from 01 January 2010 until 31 December 2012 (Themba Lethu) or 15 October 2013 (Khayelitsha). Khayelitsha HIV Treatment Programme, which comprises three primary healthcare clinics, and Themba Lethu Clinic, which is based at a secondary hospital, both manage patients according to standard ART guidelines published by the National Department of Health, which follow the World Health Organization recommendations. Both sites prospectively collect routine clinical data, including demographic details, antiretroviral prescriptions, and laboratory results, electronically.[10, 11]

### South African ART guidelines

The CD4 count threshold for ART eligibility changed from <200 cells/μL to <350 cells/μL in April 2013.[12] Recommended first line ART from 2010 was tenofovir, emtricitabine or lamivudine, and efavirenz.[13]

### Data management

Anonymised data were exported from both sites’ electronic databases using a standard data transfer format. We checked for data inconsistencies and possible errors, which were sent to the sites for resolution. In addition, we selected a stratified random sample (stratified by sex, ART regimen, year of ART start, and baseline tuberculosis and pregnancy status) of 25% of patients overall for detailed checking of ART prescription data against clinic records, and to identify reasons for antiretroviral discontinuations where those were missing, through retrospective folder reviews where required (‘the enhanced-data sample’). The routine-data group comprised all patients not included in the enhanced-data sample.

### Analysis

We defined antiretroviral discontinuations as discontinuation of at least one first-line antiretroviral drug for any reason, regardless of whether or not another drug was substituted. Reasons for discontinuations were determined at the time of discontinuation by the treating clinician, and included adverse drug reactions, treatment failure, contraindication (including pregnancy and drug interactions), or guideline change. Causality assessment of adverse drug reactions was based on clinical judgement of the treating clinician. In addition, discontinuations of at least two drugs (including a switch from a non-nucleoside reverse transcriptase inhibitor to a protease inhibitor), after at least one viral load >1000 copies/mL, that occurred at least six months after treatment initiation were also classified as treatment failures, provided that no other reason was given by the treating clinician.

We estimated the incidence of antiretroviral discontinuations for the five most-commonly prescribed antiretrovirals using Kaplan-Meier analyses. We estimated the drug-specific incidence of discontinuations for different reasons using competing risks analysis. We explored associations between discontinuations due to adverse drug reactions and treatment site, sex, age, and baseline weight, CD4 count, and estimated glomerular filtration rate using competing risks regression. Antiretroviral discontinuations for reasons other than toxicity were considered competing risks. Patients were censored at death, loss to follow up, transfer to another treatment facility, or analysis closure (six months before database closure at each site). Patients were considered lost to follow up if they had no clinic visit after analysis closure (six months before database closure).

### Ethics

The University of Cape Town and the University of the Witwatersrand Human Research Ethics Committees approved the sites’ contribution of anonymised data to the study. The University of Cape Town Human Research Ethics Committee approved the analysis. The study protocol was also reviewed and cleared as human subjects' research by the U.S. Centers for Disease Control and Prevention (CDC).

## Results

### Study cohort

We included 15 396 patients: 9 630 from Khayelitsha, and 5 766 from Themba Lethu. Their characteristics at the time of ART initiation are summarized in Table 1. The characteristics of the 3 837 patients in the enhanced-data sample were similar to those in the routine-data group (S1 Table). The most common first-line ART regimen was tenofovir, lamivudine, and efavirenz (73.9%), followed by stavudine, lamivudine, and efavirenz (10.4%), and tenofovir, lamivudine, and nevirapine (8.4%). The numbers of patients who received each antiretroviral are shown in Table 1. Median duration of follow up was 12.7 months (interquartile range (IQR) 5.2 to 22.9). By the end of follow up 11 480 (75%) remained alive and in care at the sites, 2 580 (17%) were lost to follow up, 671 (4%) had transferred to other clinics, and 663 (4%) had died.

**Table 1:** Characteristics at first-line antiretroviral treatment initiation of adult patients from two South African cohorts, 2010–2013.

	All patients	Khayelitsha	Themba Lethu
n	15 396	9 630	5 766
Males, n (%)	5 669 (37)	3 411 (35)	2 258 (39)
Mean age in years (SD)	36.7 (±9.1)	35.6 (±8.8)	38.7 (±9.4)
Median CD4 count in cells/μL (IQR)	165 (84 to 238)	179 (98 to 250)	144 (68 to 219)
Pregnant, n (percentage of women)	484 (5)	424 (7)	60 (2)
Current tuberculosis, n (%)	3 110 (20)^1^	2 400 (25)^1^	710 (12)
First line antiretrovirals, n (%):			
Abacavir	41 (0.2)	2 (0.02)	39 (1)
Zidovudine	611 (4)	463 (5)	148 (3)
Stavudine	1 857 (12)	764 (8)	1 093 (19)
Tenofovir	12 888 (84)	8 401 (87)	4 487 (78)
Efavirenz	13 462 (87)	8 177 (85)	5 285 (92)
Nevirapine	1 695 (11)	1 379 (14)	316 (5)
Lopinavir (ritonavir-boosted)	237 (2)	74 (1)	163 (3)
Atazanavir	2 (0.01)	0	(0.03)

1. Tuberculosis status unknown in 70 patients.

IQR: interquartile range; SD: standard deviation.

### Antiretroviral discontinuations

Overall 1 832/15 396 patients (12%) had an antiretroviral discontinuation. The incidence of antiretroviral discontinuation for any reason was 110.3 per 1 000 patient years (95% confidence interval (CI) 105.3 to 115.4). Of those remaining in care for at least three years, 298/495 (60%) remained on their initial regimen at three years. This differed according to ART regimen: 81/99 (82%) of those on any tenofovir-based regimen (predominantly combined with efavirenz and either lamiviudine or emtricitabine), and 128/262 (49%) of those on any stavudine-based regimen were still on their original regimen at three years (risk difference 33%, 95% CI: 23 to 43%).

### Reasons for antiretroviral discontinuations

The reason for antiretroviral drug discontinuation was unknown in 35% of patients in the routine-data group, and 16% in the enhanced-data sample (after reasons for antiretroviral discontinuations were checked). The incidence of antiretroviral discontinuations due to ADRs was 28.2 (95% CI 25.4 to 31.3) per 1 000 patient years in the routine-data group, and 45.9 (95% CI: 39.8 to 52.9) per 1 000 patient years in those patients in the enhanced-data sample.

**Fig 1:**
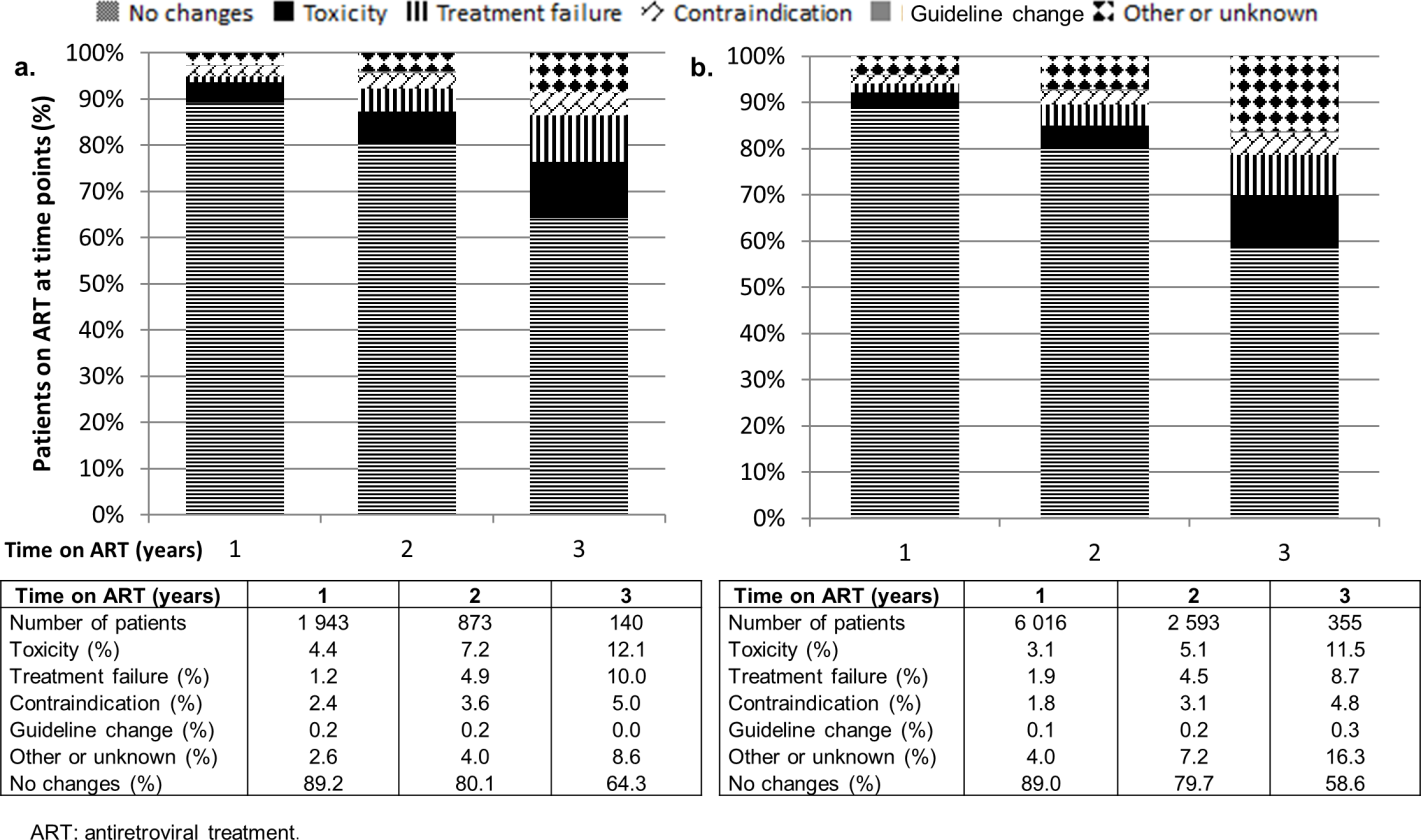
Antiretroviral discontinuations amongst a. enhanced-data sample patients (n = 3 837) and b. routine-data patients (n = 11 559) from two South African cohorts, who initiated antiretroviral treatment from 2010–2013 (denominator is those who remained alive and in care for 1, 2, and 3 years after ART initiation respectively).

In the enhanced-data sample ADR was the most common reason for antiretroviral discontinuation, followed by treatment failure: 12.1% and 10.0% of patients had an antiretroviral discontinuation because of toxicity or treatment failure respectively by 3 years on ART. The proportion of antiretroviral discontinuations (amongst enhanced-data sample patients still alive and in care at 1, 2, and 3 years) attributable to the various causes is shown in Fig 1A. In patients in the routine-data group, the relative ranking of causes for antiretroviral discontinuations was the same, although the proportions were lower for known causes, and unknown causes comprised a much larger proportion (Fig 1B.)

### Antiretroviral discontinuations due to ADRs in the enhanced-data sample

**Fig 2:**
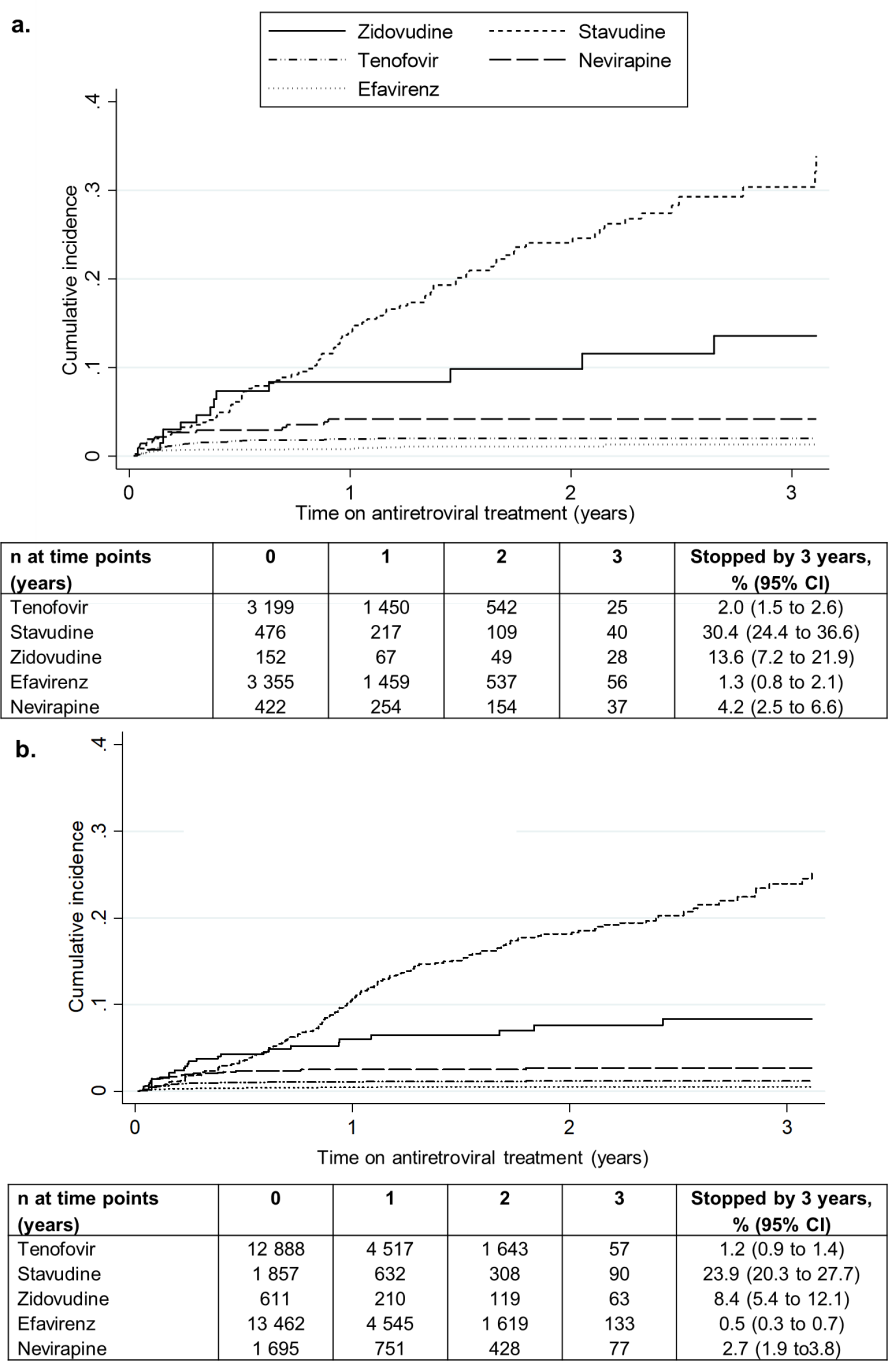
Cumulative incidence of antiretroviral discontinuations due to ADRs according to antiretroviral amongst a. enhanced-data sample patients (n = 3 837) and b. routine-data patients (n = 11 559) from two South African cohorts, who initiated antiretroviral treatment from 2010–2013.

The cumulative incidence of antiretroviral discontinuations due to ADRs according to each antiretroviral is shown in Fig 2A for patients in the enhanced-data sample. Although the pattern of discontinuations was similar in the routine-data group, the absolute incidence was underestimated (Fig 2B).

The majority of tenofovir discontinuations due to ADRs were for kidney toxicity (38/54, 70%). In a further 11 patients (20%) kidney toxicity was the likely reason, as they had a decrease in estimated glomerular filtration rate (eGFR) (in 8 patients to less than 60 mL/min) before tenofovir was discontinued.

Stavudine discontinuations due to ADRs were for lipodystrophy (34/84, 40%), peripheral neuropathy (20/84, 24%), and hyperlactataemia (11/84, 13%); the toxicity was unspecified in 11 patients. The majority of zidovudine discontinuations due to ADRs were for haematological toxicity (9/13 patients).

Efavirenz discontinuations due to ADRs were for hypersensitivity (11/29, 38%), neurological/neuropsychiatric toxicity (10/29, 34%), and hepatotoxicity in 1 patient. Nevirapine discontinuations due to ADRs were for hypersensitivity (9/16), or hepatotoxicity (7 patients).

### Predictors of antiretroviral discontinuations due to ADRs

In a multivariable analysis of the enhanced-data sample patients, tenofovir, stavudine, and nevirapine discontinuations due to ADRs were associated with treatment site (Table 2A). In addition, tenofovir discontinuations were associated with older age, male sex, and lower baseline CD4 count, weight, and eGFR. Stavudine and zidovudine discontinuations were associated with female sex. None of the predictor variables that we considered was associated with efavirenz discontinuations. In the routine data group, associations between treatment discontinuations and the variables listed above were broadly similar to those seen in the enhanced-data sample (Table 2B).

**Table 2:** Associations with discontinuations due to adverse drug reactions in a. enhanced-data patients (n = 3 837) and b. routine-data patients (n = 11 559) from two South African cohorts, who initiated antiretroviral treatment from 2010–2013.

**a.**	**Tenofovir**		**Stavudine**		**Efavirenz**		**Nevirapine**		**Zidovudine**	
n (in multivariable model)	2 217		353		2 341		305		99	
Number of discontinuations due to ADRs	37		67		20		12		8	
	**SHR**	**95% CI**	**SHR**	**95% CI**	**SHR**	**95% CI**	**SHR**	**95% CI**	**SHR**	**95% CI**
Themba Lethu site (reference: Khayelitsha)	**19.8**	**6.4 to 61.5**	**3.1**	**1.7 to 5.7**	1.8	0.7 to 4.3	**9.7**	**1.8 to 51.1**	2.7	0.5 to 16.4
Baseline age (per 10 year increase)	**1.6**	**1.1 to 2.3**	1.0	0.8 to 1.3	1.3	0.8 to 2.1	1.6	0.7 to 3.6	0.5	0.2 to 1.3
Baseline CD4 count (per 50 cells/μL decrease)	**1.2**	**1.0 to 1.4**	1.1	1.0 to 1.3	1.0	0.8 to 1.3	1.0	0.7 to 1.5	1.4	0.8 to 2.3
Baseline weight (per 10 kg decrease)	**1.9**	**1.3 to 2.6**	1.1	0.9 to 1.3	1.0	0.7 to 1.4	0.9	0.6 to 1.2	1.0	0.7 to 1.5
Baseline eGFR (per 10 mL/min decrease)	**1.5**	**1.3 to 1.7**	1.0	0.9 to 1.1	1.1	0.8 to 1.4	1.2	0.9 to 1.7	1.0	0.7 to 1.3
Female (reference: male)	**0.4**	**0.2 to 0.6**	**2.5**	**1.4 to 4.2**	1.1	0.4 to 3.1	1.4	0.2 to 8.9	**0.2**	**0.0 to 0.9**
**b.**	**Tenofovir**		**Stavudine**		**Efavirenz**		**Nevirapine**		**Zidovudine**	
n (in multivariable model)	6 796		989		7 098		878		283	
Number of discontinuations due to ADRs	78		140		35		24		15	
	**SHR**	**95% CI**	**SHR**	**95% CI**	**SHR**	**95% CI**	**SHR**	**95% CI**	**SHR**	**95% CI**
Themba Lethu site (reference: Khayelitsha)	**22.5**	**9.1 to 55.6**	**1.9**	**1.3 to 2.8**	**2.8**	**1.4 to 5.6**	1.9	0.7 to 5.0	2.9	0.8 to 10.7
Baseline age (per 10 year increase)	**1.2**	**0.9 to 1.5**	1.1	0.9 to 1.3	1.2	0.8 to 1.7	0.7	0.3 to 1.4	0.7	0.4 to 1.1
Baseline CD4 count (per 50 cells/μL decrease)	**1.2**	**1.1 to 1.4**	1.0	0.9 to 1.1	0.9	0.8 to 1.0	1.0	0.8 to 1.3	1.0	0.8 to 1.1
Baseline weight (per 10 kg decrease)	**1.8**	**1.4 to 2.3**	0.9	0.8 to 1.0	1.1	0.9 to 1.4	1.3	1.0 to 1.8	1.2	0.9 to 1.8
Baseline eGFR (per 10 mL/min decrease)	**1.3**	**1.2 to 1.5**	1.0	0.9 to 1.1	1.0	0.9 to 1.2	1.0	0.9 to 1.2	1.2	0.9 to 1.5
Female (reference: male)	**0.5**	**0.3 to 0.8**	**2.2**	**1.5 to 3.2**	1.4	0.6 to 3.0	0.4	0.1 to 1.3	0.7	0.2 to 2.9

CI: confidence interval; eGFR: estimated glomerular filtration rate; SHR: sub hazard ratio. SHRs adjusted for the other variables in the model. Significant associations are indicated in bold type.

## Discussion

In our cohort of 15 396 patients, 40% had a first-line antiretroviral discontinuation by three years on ART. In the enhanced-data sample of 3 837 patients, we were able to determine the reason for 84% of discontinuations. ADR was the most common reason for antiretroviral discontinuations, followed by treatment failure, with 12% and 10% of patients having discontinued antiretrovirals for those reasons respectively by three years on ART. The incidence of discontinuations differed by antiretroviral drug: 30% and 2% of patients had stopped stavudine or tenofovir respectively because of ADRs by three years on ART. Although the relative rankings of antiretroviral discontinuations were similar using routine data only, lack of additional checking for reasons resulted in underestimates of the incidence of antiretroviral discontinuations due to ADRs. Standard first line antiretrovirals tenofovir and efavirenz had the lowest incidences of discontinuations due to ADRs. The majority of tenofovir discontinuations were due to decreases in kidney function, and the majority of efavirenz discontinuations were due to hypersensitivity reactions or neuropsychiatric symptoms. Associations with tenofovir discontinuations due to ADRs included older age, and lower baseline CD4 count, weight, and estimated glomerular filtration rate. We found no associations between efavirenz discontinuations due to ADRs and potential risk factors that we considered. In general, discontinuations for ADRs were more likely to occur at the hospital-based site (Themba Lethu) than the primary health care clinic (Khayelitsha).

We found an incidence of discontinuations due to ADRs of 45.9 (95% CI 39.8 to 52.9) per 1 000 patient years. This is slightly lower than estimates from other African cohort studies that were conducted before the 2009 World Health Organization guideline change that replaced stavudine with tenofovir as part of the preferred first-line ART regimen. A study in Cote D’Ivoire reported an incidence of toxicity-related treatment modifications or interruptions of 5.2 (95% confidence interval 4.6 to 5.8) per 100 patient years.[7] A study in Kenya reported an overall incidence of treatment modifications or interruptions of 13.3 (95% CI 12.7 to 14.1), of which 46% were due to toxicity. Those antiretroviral discontinuation incidence rates are all much lower than those reported from cohort studies in Europe or the United States,[14–18] presumably in part because treatment options are more limited in lower income countries, so clinicians are less able to change antiretrovirals for relatively minor adverse events. Interestingly, while the rate of efavirenz discontinuations due to ADRs seen in our study is similar to that found in a South African study from 2007, the rate of stavudine discontinuations has increased, probably related to the more recent availability of tenofovir. As seen in previous South African studies we found a much lower rate of treatment discontinuations with tenofovir than with stavudine.[9, 19]

Using only routine data identified relative trends in antiretroviral discontinuation rates and identified relevant risk factors for antiretroviral discontinuations due to ADRs; but substantially underestimated the absolute incidence of discontinuations due to ADRs. The retrospective data checking process was labour intensive, and involved a substantial amount of time, both for generating queries, and for site staff to perform folder reviews, but reasons that were not recorded in the database were frequently available in the folders or other patient records. This suggests that improving prospective data capture systems in routine databases would increase the capacity of routine data to answer pharmacovigilance questions.

Patients at Themba Lethu, the hospital-based cohort, were more likely to have tenofovir and stavudine discontinuations due to ADRs than patients at the Khayelitsha cohort primary health care clinics. Patients at the hospital site had more frequent laboratory monitoring, and healthcare workers there were more likely to have access to blood results sooner, which might in part explain the difference. These differences in the patterns of reasons for antiretroviral discontinuations highlights the importance of using data from several representative sites in order to make conclusions regarding ADR-related antiretroviral discontinuations.

Our study had several limitations. Data were from routine clinical practice, and even after thorough data checks there were still missing data, including unknown reasons for antiretroviral discontinuations. Reasons for discontinuations were as recorded by the treating clinician. We might have underestimated the rates of ADR-related discontinuations if the reason wasn’t adequately recorded. We assessed only the first antiretroviral discontinuation, so might have underestimated rates of discontinuations that occurred later on in the course of treatment. Similarly, we might have underestimated antiretroviral discontinuations owing to patients being lost to follow up.

## Conclusions

Currently recommended first-line antiretrovirals were relatively well-tolerated in our setting.

The routine data used in our study was adequate to estimate relative trends in, and identify potential risk factors for, antiretroviral discontinuations due to ADRs. But obtaining more accurate incidence estimates involved a time- and labour-intensive data-checking process. Observational cohorts are a useful source of pharmacovigilance data. Improving prospective data capture systems would increase the capacity of routine clinical data to answer pharmacovigilance questions.

## Supporting information

S1 Table Characteristics at first-line antiretroviral treatment initiation of adult patients from two South African cohorts, 2010–2013: enhanced-data sample and routine-data group. (PDF)
