# Supplementary material for: Routine data underestimates the incidence of first-line antiretroviral drug discontinuations due to adverse drug reactions: Observational study in two South African cohorts
Source: PLoS One. 2018 Sep 5;13(9):e0203530. doi: 10.1371/journal.pone.0203530 (PMC6124775; doi:10.1371/journal.pone.0203530)
Supplement: S1 Table — (PDF) [file pone.0203530.s001.pdf]

**S1 Table: Characteristics at first-line antiretroviral treatment initiation of adult patients from two South African cohorts, 2010–2013: enhanced-data sample and routine-data group**

| | Khayelitsha | | Themba Lethu | |
|---|---|---|---|---|
| | **Enhanced-data sample** | **Routine-data group** | **Enhanced-data sample** | **Routine-data group** |
| n | 2367 | 7263 | 1470 | 4296 |
| Men, n (%) | 844 (36) | 2 567 (35) | 582 (40) | 1 676 (39) |
| Mean age in years (SD) | 35 (±9) | 36 (±9) | 39 (±9) | 39 (±9) |
| Median CD4 count in cells/µL (IQR) | 180 (99 to 249) | 178 (97 to 251) | 149 (76 to 223) | 143 (65 to 219) |
| Pregnant, n (percentage of women) | 107 (7) | 317 (7) | 17 (2) | 43 (2) |
| Current tuberculosis, n (%)[1] | 593 (25) | 1 807 (25) | 177 (12) | 533 (12) |
| First line antiretrovirals, n (%): | | | | |
| Abacavir | 0 | 2 (0.03) | 10 (1) | 29 (1) |
| Zidovudine | 115 (5) | 348 (5) | 37 (3) | 111 (3) |
| Stavudine | 192 (8) | 572 (8) | 284 (19) | 809 (19) |
| Tenofovir | 2 070 (87) | 6 341 (87) | 1 139 (77) | 3 348 (78) |
| Efavirenz | 2 005 (85) | 6 172 (85) | 1 350 (92) | 3 935 (92) |
| Nevirapine | 341 (14) | 1 038 (14) | 81 (6) | 235 (5) |
| Lopinavir (ritonavir-boosted) | 21 (1) | 53 (1) | 39 (3) | 124 (3) |
| Atazanavir | 0 | 0 | 0 | 2 (0.05) |

1. Tuberculosis status unknown in 70 patients. IQR: interquartile range); SD: standard deviation
